# Ethical considerations in Controlled Human Malaria Infection studies in low resource settings: Experiences and perceptions of study participants in a malaria Challenge study in Kenya

**DOI:** 10.12688/wellcomeopenres.14439.2

**Published:** 2018-10-29

**Authors:** Maureen Njue, Patricia Njuguna, Melissa C. Kapulu, Gladys Sanga, Philip Bejon, Vicki Marsh, Sassy Molyneux, Dorcas Kamuya

**Affiliations:** 1 KEMRI-Wellcome Trust Research Programme, Kilifi, Kenya; 2 Institute of Tropical Medicine, Antwerp, Belgium; 3 World Health Organisation, Geneva, Switzerland; 4 Centre for Tropical Medicine and Global Health, Nuffield Department of Medicine, University Oxford, Oxford, OX3 7BN, UK

**Keywords:** Ethics, CHMI Volunteer Infection studies, risks, informed consent, malaria, developing countries

## Abstract

**Background:** The range and amount of volunteer infection studies, known as Controlled Human Infection Model (CHMI) studies, in Low-Middle Income Countries (LMICs) is increasing with rapid technological advancement, world-class laboratory facilities and increasing capacity development initiatives. However, the ethical issues these studies present in LMICs have not been empirically studied. We present findings of a descriptive social science study nested within a malaria volunteer infection study, on-going at the time of writing, at the KEMRI-Wellcome Trust Research Programme (KWTRP) on the Kenyan Coast.

**Methods:** The study included non-participant observations, five group discussions with more than half of the CHMI study participants, two in-depth interviews with study team members, and an exit questionnaire administered to the participants.

**Results:** Participants understood the key elements of the study, including that they would be deliberately infected with malaria parasites and may get malaria as a result, there would be regular blood draws, and they would spend up to 24 days in a residence facility away from their homes. The greatest motivation for participation was the monetary compensation of 20 USD per overnight stay given as a lump-sum at the end of their residency stay. Also appreciated were the health screening tests prior to enrolment and the positive relations with the study team. Concerns raised included the amount and regularity of blood draws experienced, and concerns that this type of research may feed into on-going rumours about research generally.

**Conclusion: **With the increasing range and number of CHMI studies being conducted in LMICs, current ethical guidance are inadequate. ****This study highlights some of the ethical issues that could emerge in these settings, emphasizing the heavy responsibility placed on research review and regulatory systems, researchers and funders, as well as the importance of carefully tailored community engagement and consent processes.

## Introduction

Controlled Human Malaria Infection (CHMI) studies – also referred to as volunteer infection or Challenge studies - involve the deliberate infection of healthy volunteers with malaria parasites to assess the efficacy of potential vaccine and drug candidates and to understand the innate and acquired protection against malaria parasites ^1, 2^. Vaccine development is a lengthy, complex and resource intensive process and CHMI studies in endemic populations are therefore being proposed to hasten the identification and development of potential vaccine candidates by studying natural immunity. CHMI studies have been conducted in non-malaria endemic areas to provide a faster and more cost-effective way of testing vaccine candidates compared to large scale clinical trials involving numerous human subjects ^1, 3, 4^.

Few CHMI studies have been conducted in Africa for reasons such as: lack of supportive infrastructure to produce and store infectious material that would be used in the healthy volunteers; inadequate clinical trial facilities that would be necessary for these types of studies; and inadequate expertise to conduct such studies safely ^1, 2^. However, these limitations have started to be addressed ^2, 5– 7^. In addition, the development of aseptic, purified, cryopreserved, infectious *Plasmodium falciparum* sporozoites (PfSPZ) for infection, also referred to as PfSPZ challenge, enables Challenge studies to be conducted in areas where infectious mosquitoes would be difficult to produce or import ^1, 2, 8^. In the last 6 years, at least 7 CHMI studies have been conducted in Africa: in Tanzania ^2^, Kenya ^8^, and Gabon ^1^; with 4 on-going in Kenya (ClinicalTrials.gov; NCT02739763), Equatorial Guinea ( NCT02859350), and Mali ( NCT02996695; NCT02627456) ^9^.

CHMI studies promise to accelerate vaccine development, but the ethical issues need careful consideration, particularly in contexts where the population might have low exposure to scientific elements of research, vast unmet health needs, constrained health care systems and in cases where many families are struggling socio-economically. The intentional infection of healthy volunteers with a disease-causing pathogen has the potential to raise concerns among the public who generally do not expect this of medicine and medical research ^4, 10, 11^. It is therefore particularly important to ensure that such studies are conducted within well considered, transparent guidelines and regulatory processes ^11^, and also that any discomfort associated with the infection is appropriately addressed ^4^. Challenge studies also often do not directly benefit the individual medically, although there may be an indirect benefit from health screening and medical care. Rather, the benefit is at societal level through scientific innovation and improved public health ^4, 12^. These societal benefits must be balanced against protecting participant rights and interests ^4, 12^.

Challenge studies often require participants to stay in in-patient settings to allow close monitoring of safety, prevent infection to others, and – sometimes - the participant’s environment to be controlled ^1, 4^. The time lost through in-patient stays can significantly inconvenience participants and prevent them from engaging in their usual activities ^1^. Individuals without stable jobs or who are economically underprivileged might be disproportionally attracted, raising concerns about potential exploitation ^4^. Relatedly, although it is recognized that participants should be appropriately compensated for inconvenience and lost wages, payments should not ‘unduly influence’ participants, such that they do not carefully consider the potential risks and discomforts, or even conceal relevant medical history to maximize chances of participation ^4^.

For many of the above reasons, information requirements for Challenge studies are often complex and long. Researchers therefore often target participants with higher levels of education as most likely to give *informed* consent ^1, 2, 8^. However, this might introduce a new dilemma of excluding those with low formal education. Another consent related issue for Challenge studies is that some studies may condition or limit the right to withdraw for the individual participants’ (or others’) safety. Miller and Grady have argued that while limitations on freedoms could be restricted to eliminate these risks, their right to withdraw from further study procedures should be respected ^4^. Investigators should consider in advance the processes to follow (for instance the provision of emergency treatment) should a participant abruptly express a wish to leave. Persuasion may be justifiable where participant safety is an issue, but coercion to maintain participation in research must be avoided, and deprivation of liberty is never an option ^4^. In this article we present one of the first studies from a Low-Middle Income Country (LMIC) exploring the experiences and perceptions of participants in a Challenge study. We discuss the ethical issues emanating from the participants’ involvement and consider the implications for conducting CHMI studies in LMICs.

## Methods

### Study context

KEMRI-Wellcome Trust Research Programme (KWTRP) ^1^, where the CHMI study is being conducted, is a long-standing internationally recognized health research programme in Kenya with its headquarters on the Kenyan Coast (Kilifi), and offices in Nairobi (Kenya) and Mbale (Uganda). A range of multi-disciplinary research relevant to local, national and regional needs and priorities is undertaken across these sites. This research spans four broad scientific themes: vaccines; genomics and infectious disease transmission; clinical research; and health systems and research ethics. World-class laboratory facilities with the latest technology, and a vibrant community engagement platform ^13^ support the research activities undertaken at the Programme. An integrated Kilifi Health and Demographic Surveillance System has been running for over 15 years involving over 280,000 residents living in around the Kilifi County Hospital ^14^ so as to recruit participants from a range of malaria transmission settings. A collaborative working arrangement with the County Hospital management has made possible long-term strategic support in health facilities, and research is integrated into the health care system. The Kilifi CHMI participants were drawn from specific locations of the KHDSS. All studies conducted by the Programme are approved by local, national and sometimes international scientific and ethics review committees.

### The Controlled Human Malaria Infection study in Kenya

The current CHMI study in Kenya follows on from a previous CHMI study that we conducted in Nairobi in 2012 ^8^. The aim of the current CHMI study is to assess human immunity to *P. falciparum* using sporozoites (PfSPZ Challenge) administered by direct venous inoculation. The study intends to screen 2000 individuals and eventually enroll 200 participants (aged between 18 and 45 years) with prior exposure to malaria and varying levels of immunity from three sites - western Kenya (Ahero), coastal Kenya (Kilifi) and central Kenya (Nairobi) (ClinicalTrials.gov; NCT02739763). The study so far has included three challenge events. Two of the three challenge events at the Kilifi site have been conducted and completed involving 101 participants with the third currently ongoing. The social science study was built around the 2 ^nd^ challenge event. 114 participants were screened in the 2 ^nd^ Challenge event for eligibility at the KWTRP; 64 (49 male; 15 female) participants were enrolled in to the study. Table 1 below summarizes the CHMI study procedures.

**Table 1.** The Controlled Human Infection Model (CHMI) study procedures.

Stage	Procedures
Pre-screening	• Community and stakeholder engagement • Information giving sessions (several sessions) • Seeking consent; • Test of understanding (only those who pass are enrolled)
Screening	• Pulse, blood pressure, respiratory rate and temperature measurements taken About 20mls mls blood sample taken for the following laboratory assays • Haematology: Full Blood Count, screen for sickle cell trait. • Biochemistry: Sodium, Potassium, Urea, Creatinine, Albumin, ALT and bilirubin. • Diagnostic serology: HIV antibodies, Hepatitis B. • Immunological assays of prior exposure to malaria • Diagnostic Malaria Tests Urinalysis, and for women pregnancy test Electrocardiograms (ECGs) for evidence of heart disease. Medical and social history and clinical assessment
Day before Challenge (C-1)	• 59 mls venous blood sample for repeat of screening tests • Clinical assessment of any new medical issues or symptoms; and including height and weight measurements, • Urine analysis for women to determine if pregnant • Enrolment into in-patient facility
Challenge day	• Pulse, blood pressure, respiratory rate and temperature measurements taken • 3,200 parasites injected intravenously • Volunteers observed for 1 hour after injection before returning to in-patient facility (at Pwani University)
Day 5 post challenge	• 32 mls of blood sample for immunological analyses
Days 1–6 Post Challenge	• Volunteers in residence, presence of clinical staff throughout to monitor for adverse events
Days 7–14 Post Challenge	• Clinical assessment; Volunteers asked about any symptoms of malaria • Venous blood samples (each 4ml) taken twice daily (i.e. morning and evening) for PCR for *P. falciparum* • In addition, on day 7 ^th^, 9 ^th^ and 14 ^th^, additional blood volumes are taken; 33mls each on day 7 ^th^ and 14 ^th^ and 32 mls on day 9 ^th^ for immunological assays • 2 ml blood sample taken at 9 ^th^ day for biochemistry
Day 15–21 post challenge	• Once daily venous blood sample of 4mls • Clinical assessment
If diagnosed with malaria at any time	• 41ml Blood sample for various tests • Start anti-malaria treatment; For three days, observation, once daily blood sample (4mls) taken to check clearance of parasites • 5mls blood sample taken at 72 hours to check if parasites cleared and full blood count; then discharged and reminded of post-35 day follow-up visit
Day 35 Post Challenge	• All volunteers reviewed in the nearest clinic • Clinical assessments performed and AEs assessed. • Venipuncture performed (51 mls of blood) for immunological assays and full blood count

The enrolled participants were in residence for an average of 18 days (range 15 – 24 days) at a guesthouse within Pwani University (a local university about 2.5km away from KWTRP). The length of residence depended on the time to meet the criteria for treatment of malaria (at which point they were treated for 3 days and then discharged when clear of parasites); or treatment at day 21 because of not reaching the set criteria. The three-day course of anti-malaria drug (the recommended artemether-lumefantrine) was administered by the clinical team and directly observed. A total of 412mls of blood per individual was drawn over a period of 3 months.

*Community and stakeholder engagement* was undertaken prior to and throughout the CHMI study. This included information sharing sessions with key stakeholders - hospital administration, health facility staff, local administrative leaders (chiefs and assistant chiefs), Pwani-University Administration, and with KEMRI-Community Representatives – a network of about 220 people elected by the local residents to consult on research activities ^15^. Barazas ^2^ were used to provide general information about the study to the population in the three sub-locations where the Kilifi participants were recruited from. Interested adults were invited for further information giving sessions at the nearest health facility. At the health facility, the study clinician further explained the study to groups of up to 15 potential participants using information in consent forms *(see Supplementary File 1*); followed by one-on-one sessions with each interested potential participant for clarification of any questions. The potential participants were then invited to undergo a test of understanding ( *see Supplementary File 2*), where they were required to get all 9 questions correct in two attempts. Two people did not pass the test and therefore were not enrolled into the study. Information continued to be provided to enrolled participants throughout the study. Further engagement with participants facilitated by the Community Liaison Group (CLG) ^3^ continued while in residence, and included an open day at the Research Programme, a tour of the Labs, and talks with researcher and with CLG members.

### Social science sub-study

The social science study was nested in the 2 ^nd^ Challenge event at the Kilifi site and was undertaken between January and April 2017. The social scientists were not part of the study team but worked closely with the study team. They were introduced to the participants by the study team. MN spent considerable time with CHMI participants and the study team members to build good rapport given the sensitivity of the study, and to be familiar with the study procedures. She observed information giving sessions, screening and all Challenge procedures. The data from the observations provided insight into the activities of the trial and facilitated the development of the interview and FGD question guides. Interviews were conducted between 7 ^th^ – 14 ^th^ days post-challenge: Two focus group discussions (FGDs) with 14 female participants, three FGDs with 22 male participants, and two in-depth interviews (IDIs) with study team members were held using semi-structured topic guides ( *see Supplementary Files 3 and Supplementary Files 4*). The study participants were selected purposively to ensure diversity in views based on gender, age and education levels. The study team selection was based on convenient sampling.

 A semi-structured questionnaire was administered by clinicians to all participants attending day-35 post-challenge follow-up visit. The questionnaire data has not been included in this manuscript but will be utilized in a larger body of social science work going on within the Challenge studies at KWTRP.

All interviews were audio-recorded, transcribed, translated into English and managed using NVivo 10 software. A thematic content approach was used to analyse the data, with an iterative process of coding building into categories and themes that were then applied to the entire dataset. The analysis was primarily conducted by MN and DK with the support of the other authors in an iterative process. The themes were developed both deductively (from major themes in the interview/FGD guides) and inductively (from the emerging issues in the transcripts). Some of the themes included, informed consent processes, motivations for participation (compensation and health benefits), perceptions of the trial and the challenge model, experiences in the trial and in-patient facility and decision making and negotiations with significant others.

### Ethical review

This social science sub-study was reviewed and approved by the Kenya Medical Research Institute (KEMRI) Scientific and Ethics Review Unit ( *KEMRI/SERU/CGMRC/029/3190*) and the Oxford Tropical Research Ethics Committee (OxTREC 2-16). Written informed consent was sought from participants for all interviews (IDIS and FGD) and for audio-recording.

## Results

### Participants’ characteristics

Over half of the CHMI participants (36 out of 64) participated in the social science sub-study. Table 2 below shows the characteristics of the 36 respondents. Most of the respondents (34%) were 21–30 years old; all males had at least 5 years of schooling while 2 of the females had less than 3 years of schooling. For this particular Challenge event, participants who had very low levels of schooling could also correctly answer the test of understanding questions thus showing an understanding of the study and its procedures.

**Table 2.** Characteristics of participants in the social science sub-study.

Characteristic	Female (n=14) 39%	Male (n=22) 61%	Total (n=36)
***Age, years***			
21 – 25	4	8	12
26 – 30	4	6	10
31 – 35	3	2	5
36 – 40	2	3	5
41 – 45	1	2	3
***Level of education (years completed in school)***			
No formal Education	1	0	1
Adult education/1–2 years	1	0	1
Primary Education: 3–4 years	3	0	3
5–8 years	5	11	16
Secondary education: 9–10 years	1	1	2
11–12 years	3	10	13

* Missing age information from 1 male participant

### The informed consent processes

In all the FGDs, participants appreciated the study information provided, the processes of seeking consent followed and the many opportunities to discuss the study and ask questions. However, a participant noted that even though the clinical team was approachable and friendly, they were busy and could not always optimally respond to issues.

*[FGD4 P9: … someone being at work maybe you draw blood or are dealing with the files, and someone asks you a question…Someone answers in a rush such that you cannot understand. Seems like they have a lot of issues in their minds, it’s required that you at least set aside some time to answer the question well.] (Male; 38 years; 8 years education)*

A concern raised by participants in all the FGDs was information about blood volumes. Although they acknowledged that information had been given and informed consent documents provided, participants felt that using visuals to explain the blood volumes and their frequency would have enhanced comprehension. This suggestion was fed back to the team who immediately acted on the recommendation.

*[FGD5 P6: …the first time we were told* [about blood volume], *but most of us did not understand, but I later, I had to request for the form again, I went through it again, XXX1 [clinician] brought it to me, I saw on my side the procedures were ok, then later yesterday in a meeting still, XXX2 [another clinician] did this thing practically, at the meeting, …she brought a cup of water, a spoon and a syringe, so she measured and placed there, so we were able to verify that it was ok. So, I no longer have any doubts] (Male; 32 years; 12 years education)*
*[IDI2: I think physically seeing the tubes and the process, it would have really helped if they would have been mentally prepared, to see that this is the volume and the blood will be taken and kept in this tube, and the number of tubes will be this… if we could have really explained it in pictorial or a short film so that they know] (Study team member).*

The community engagement processes, both prior to enrolling in the study and afterwards, were highly appreciated as they addressed rumours that participants had heard about the research centre. These rumours have been widely documented ^13, 16^ and given the sensitivities of the study the study team were aware that they were likely to flare-up again, contributing to the careful community engagement process described above.

*[FGD2 P5: I am also impressed because where we come from we are told that people are bled and it’s not known where blood is taken, but when we were told we are going to the lab I was very keen to know what happens. And when I came out [of the lab tour] I was really satisfied, I am now longing to go home and have someone tell me blah! Blah!, so that I can explain to them everything that I have seen, that there is no unfairness whatsoever.] (Female; 24 years; 12 years education)*

### Participant perceptions of the study and infection model

In all FGDs, participants seemed to understand that they were taking part in a research study, they had been injected with malaria parasites and would get malaria as a result. Participants understood the aim was to study their immunity against the malaria parasites; and that the study would contribute to vaccine development.

*[FGD3 P1: … this word malaria challenge…what I understood…it’s about a malaria vaccine that is needed, so the biggest agenda for all these things that are going on is about malaria vaccine…The blood that is being taken will continue to be investigated then eventually to be able to get malaria vaccine] (Male; 39 years; 12 years education)*

Although CHMI differs from other clinical trials and intervention studies, participants were not particularly worried. Potential worries were alleviated by several factors: knowledge that malaria is curable; living in a malaria endemic area where there is much experience of having malaria, and knowing that treatment would be provided if necessary. In addition, they had seen that those in the 1st Challenge event appeared to be well; they had assurances from the study team that this type of study had been safely conducted elsewhere in Africa; and they had 24-hour monitoring by clinical staff:

*[FGD5 P4: I was injected with malaria, I haven’t been sick for four years and I don't know how serious the condition will be. However, I just volunteered and it’s because we spend the night with them here, a nurse is here day and night. I knew that if my head starts to pain I will go to her/him for them to see how they can help.](Male; 24 years; 8 years education)*

Participants said that they were not worried about being injected with the malaria parasites, but said they would be concerned about being injected with a less familiar disease-causing organism. That the latter was not happening required participants’ trust in the researchers:

*[FGD1 P8: …[my neighbour] told me, “how do you know it’s only malaria, what if you are injected with HIV”? I told her that I have been tested, I have enrolled in the study, I know I don’t have the HIV virus and I don’t have any illness, they have done a medical test, if now I get HIV I’ll know it’s them…That day when I was injected she came back to me, and told me “you have already been injected with HIV” …] (Female; 23 years; 12 years education)*

When asked if they would participate in future Challenge studies, most respondents said that they would if the illness was curable; most spontaneously mentioning that they would not participate in an HIV challenge study. 

### Motivation for research participation

Three main reasons for participating in the study were the monetary compensation (payment) provided, the health care benefits, and wanting to contribute to the health of communities.

Compensation for time for the in-patient stay was paid at a rate of 2000 Kenya Shillings (US$20) per overnight stay. Time compensation at most research studies at the Programme is based on a government daily wage rate for unskilled labour which is 350 Kenya shillings (US$3.5) per day. Although compensation was a key motivating factor for many participants to join, some participants indicated that the amounts were similar overall to their daily casual labour earnings.

*[FGD1 P8: I normally sell clothes, I did my calculations, per day if I go to the market, I sometimes make more than 3000, but sometimes, like now, there is no money You…make like 1000, sometimes you make like 800 shillings, so I…thought these 2000 shillings everyday are better, If I manage to stay for those 24 days, I will have gotten a lot more money than going to the market, so I decided to come here] (Female; 23 years; 12 years education)*

The difference in this case is the predictability of the amount per day and the lump sum payment at the end of the in-patient stay; a total of Ksh. 48,000 (USD $480). Many participants indicated that they would use this income to develop their families, pay school fees, pay-off debts, buy livestock, go for vocational training, open businesses and build houses. The participants seemed to understand that the cash provided was compensation for the time away from other productive work and the high levels of inconvenience in the study as opposed to a payment. Many had made a calculated decision based on what they stood to gain:

*[FGD2 P1: …I have a child who is finishing (Primary) school and because you cannot get all that money together at once for doing shopping for him, and if I come here I will get the cash at once, which I will use for him to start (Secondary) school, that’s why I decided to come here ...] (Female; 32 years; 6 years education)*

*[FGD5 P3: I have already set a budget for the money; every coin is allocated and if I get malaria today it will have disrupted my plans.] (Male; 25 years; 5 years education)*

The allure of the cash compensation was also expressed by those who had not been enrolled after screening in the 1 ^st^ challenge event of the study, but were enrolled for the 2 ^nd^ event. They carefully monitored progress of the study, hopeful that they would be eligible, and were disgruntled when they were not:

*[FGD1 P7: There is one [person] who got really angry… he was told since he smokes a lot he cannot participate. He called XXX1 and said, “From today I will not participate in any KEMRI study and I don’t want my children to participate in any study, from today and all the days of my life” so it’s like he got angry because he was not allowed to participate] (Female; 28 years; 12 years education)*

Although the cash compensation was a great motivator for research participation, participants were however apprehensive and uncomfortable when presented with a hypothetical increase or decrease in the level of monetary compensation offered. If there had not been any monetary compensation, many felt that they would not have participated in the study;

*[FGD3 P2: …the vaccine is important, but if you had been told that you are coming to look at the vaccine, free of charge, there is nothing you will get, there would be no people willing to join] (Male, 27 years, 8 years education)*

However, if the amount of monetary compensation had been too high, several participants mentioned that such high compensation would have introduced suspicions about the study.

*[FGD2 P3: it would give me worries [If compensation was higher]…I would be thinking, why am I being given ten thousand [USD 100], for doing what exactly? I am being taken there, what are they going to do to me to even get that ten thousand?] (Female; 36 years; 2 years Adult education)*

The second motivating influence were the study *health care including screening tests*, most of which were very expensive and not available in the public health care facilities (such as ECG, liver function tests); and the presence of clinical staff to attend to the participants throughout. A few suggested that the screening should be extended to the wider community to maximize the health benefit.

*[FGD2 P3: …I wouldn’t have been able to cater for the investigations that have been done on my body, before I was enrolled to be injected with the malaria parasites. There is a thorough investigation, I will have my heart tested, I will be told how my liver is, how my kidneys are, I will be told everything…I will not be able to do it on my own, that I look for money for this to be done? I can’t…that’s why people were very happy.] (Female; 36 years; 2 years Adult education)*

The third motivating influence, often mentioned alongside the above ones, was wanting to *contribute to science*, or to find a vaccine that would be beneficial to future generations. Other participants felt their participation was important as a way of *supporting the research Programme* and its work; to ensure it achieved its objectives which were viewed as being beneficial to the community.

*[FGD1 P7: …all the studies that have been done by KEMRI, I have never participated in even one, so when this study came it was my opportunity to participate, so that we can improve health so that was my most important reason…I did not qualify in the studies that were happening previously.] (Female; 28 years; 12 years education)*

### Negotiating participation with significant others

In this area, women generally bear greater responsibility for day to day family and child care than men ^17, 18^. Participant mothers sought help from their relatives (grandparents, siblings and spouses) to care for their children and families before joining the study. One female participant mentioned that she had to take her children out of school so they could live with her mother during the study, leading to family disagreements:

*[FGD2 P4: …trouble ensued with my mother, [she said] why should the children stop going to school? You don’t want to see money pass you by...you are willing to go get injections, you are not even sure how the disease will affect you, but you have decided, you are ready to have the children miss school…] (Female; 32 years; 3 years education)*

Some of the participants indicated that they had not disclosed to their significant others about their participation in the challenge study. This was because they felt that the prevailing rumours about the research Programme itself and the unfamiliar study they were being recruited into would most probably lead to their decisions not being respected, and might contribute to strained relations at home. A few had explained their absence from home as being away for a training seminar in Kilifi, while some who had disclosed their involvement in the study minimized the information they gave about it, especially regarding the blood sampling (due to rumours).

*[FGD5 P4: On the first day, I told them that I was going for a job interview…. I went home a few days ago where I told them there is a one-month seminar at Pwani* [University], *you won’t see me if I succeed but if I don’t succeed then you will see me here in the evening…Am now in Kilifi and we are communicating via phone call that I am attending a seminar.] (Male; 24 years; 8 years education)*

The female participants described having to discuss with their spouses before consenting to the study. For some participants who live with their parents, in their descriptions, it was clear that their parents’ opinions about research participation was highly respected even though they themselves were adults and would have been perceived able to make their own decisions.

*[FGD1 P7: ….my father was worried…when my sister was called she had to get permission from our father because she is still a young girl…so I told my father that what he had heard was not true. I informed him that there is no one who got any problem, that’s when he gave my sister permission…explained to him until he understood that’s when he allowed my sister to come.] (Female; 28 years; 12 years education)*

### Participant experiences in the study

Most participants were generally happy with the way the study was conducted and how well they were taken care of while in the study. The guesthouse where participants were in residence was rated favourably. Although the university is based in a busy part of town, the participants’ movement outside the premises was restricted to avoid contracting malaria from mosquitoes. While most of the men felt restricted within the facility, women were more positive, describing it as an opportunity to rest and relax.

*The use of contraceptives* as a requirement for participation in the study was explored only with the female FGDs. Most were comfortable with using contraceptives during the study having used them previously, and understood they were necessary to prevent pregnancies during the study which could be risky for the unborn child. However, some felt that contraceptive use was encouraged to prevent pregnancy while staying in the in-patient facility, and a few worried about the longer-term consequences:

*[FGD1 P8: I don’t have a child yet…when you use these family planning measures it may get to a point when you are trying to get a child you may have some complications [Female; 23 years; 12 years education]*

The main concern for both men and women was on the frequency and volumes of blood samples and the discomfort of the constant blood draws. *Despite this concern,* the participants were pleased with the cordial and open relationship they shared with the study clinical team who were described as approachable, friendly, and always willing to help and respond to issues. The participants appreciated the opportunity to meet new people and make friendships, including with fellow participants.

*[FGD2 P1: And this study has built a very good relation among different people…we’ve been having that close brotherhood, brotherhood which is not by blood but we’ve become one friend one brother…We didn’t know each other, but now I can call this one and sit with her or the other one and continue chatting and laughing as if we are at home.] (Female; 32 years; 6 years education)*
*[IDI 1: … we tried to keep a close relationship with the participants…we could even find time, we sit with them and chat, if we are free, they had some games out there, we’d sit with them and play a game or two, with that they become free with you and they will let you know if they have issues.](Study team member)*

## Discussion

The range and amount of CHMI studies being conducted in LMIC settings is increasing with rapid technological advancement, world class laboratory facilities and increasing capacity development initiatives ^1, 2, 8^. However, the ethical issues that these studies present in these contexts have not been empirically studied. This article presented findings of a descriptive social science study embedded within an on-going malaria Challenge study on the Kenyan Coast. Here we discuss several ethical issues emerging from the findings.

### Risks and burdens in Challenge studies

There are debates on the level of risk involved in challenge studies, particularly when conducted in settings endemic for the pathogen under investigation ^11^; a debate that is likely to intensify with the increasing number of Challenge studies in LMICs. Research entails some risk to participants, which may range from minimal risk (where delegated review may be considered by IRBs) ^19, 20^ through to high levels of risk, e.g. when testing potential therapeutic agents against serious disease. Minimal risks have been defined by Evers *et al*. (2015), as risks that would expose individuals to no greater likelihood of insult or injury than those encountered in everyday life or in a routine medical or psychological examination ^21^. Phase I studies and Challenge studies must minimize risk, since they enrol healthy participants and include considerable levels of discomfort and inconveniences ^3, 4^. Discomforts may be experienced during study procedures such as during blood draws; burdens and inconveniences may include time taken up by study activities such as being away from family. Measures to minimize risks and harms include a rigorous ethics review, strong emphasis on ensuring appropriate research design, trained personnel to conduct study procedures, appropriate levels of compensation for burdens and inconvenience, and maximizing the social value of the research to science ^11, 19^. In addition, it is imperative that the participants understand all the key elements of the study, including types and levels of risks and benefits. Participants in the social science study seemed to understand these key elements of the CHMI study, and that there would be no immediate therapeutic benefits directly related to the study.

Deliberately infecting healthy volunteers with a disease-causing pathogen has been described as a potential moral dilemma particularly for clinician researchers whose primary responsibility is to cure rather than cause disease ^11^. This practice can also potentially damage or ruin the reputation of doctors if the participants and wider community do not understand the reasons for such a study and the safety procedures in place ^11^. While in our study we did not specifically explore this risk, discussions with participants and study team members did indicate that injecting people with the malaria parasite was unfamiliar. It was discussed alongside on-going rumours about the research Programme in the community and may have contributed to some participants not informing their relatives about their participation. Participants’ concerns about study safety also contributed to their active surveillance of the well-being of participants who had participated in the previous Challenge events, seeing that they were well seemed to allay some concerns. Hope and MacMillian (2004) have noted that deliberately infecting health volunteers could also undermine the reputation of the health sector ^11^. Wider reputational impacts of this study is an area that we are further investigating in the on-going Challenge study.

### Community engagement and consent processes

Challenge studies often target participants with higher levels of education to ensure comprehension of the complex information ^1, 2, 4, 8^. Those we interviewed had mixed education levels including several who had not attended school but who passed the test of understanding administered before enrolment into the study. Our study shows that in our setting, high education levels might be an unnecessary exclusionary criterion, given the participant’s ability to grasp and understand the information despite their lower education levels. Rethinking this criterion becomes important, in this and similar settings where the targeted pool in the general population has low education levels. A series of steps of community engagement and consent processes were followed by the Study team, which strengthened information sharing, provided forums for questions and answers, and gave opportunities for potential participants to consult widely before making a final decision. Empirical studies have shown that ensuring participants can access information and ask questions in a range of different contexts enhances retention of information and comprehension ^1, 4^. Participants in challenge studies spend significant time at the in-patient setting, offering a great opportunity for the study team to build relationships, strengthen communication, and reiterate study information as well as other health related information. Given the necessarily complex nature of information that needs to be covered in CHMI consent forms, innovative ways for seeking and enhancing understanding of the information should be explored. In this case, the use of visual aids to demonstrate volume of blood that would be drawn, and the regularity of blood draws was appreciated once it was introduced into the information giving sessions.

### Levels of compensation in challenge studies

We found that financial compensation was one of the strongest motivations for participating in the study. Given recognized concerns about balancing appropriate compensation against undue risk ^22– 24^, the CHMI study team drew on guidelines in the programme which were developed in consultation with community representatives and consulted widely within the research programme regarding the levels and types of compensation to provide. The daily amount provided was in line with average earning for the local community to whom the study was relevant, as was reported in the interviews. One specific ethical concern related to levels of compensation, but not yet explored in CHMI studies, is the potential for interest in financial compensation to crowd out other important research information such as risks of the research ^24^. However, in this study, various information sharing sessions and community engagement processes appeared to minimise this issue. In addition, the test of understanding appeared to ensure that those enrolled in the study understood its key elements. Another specific ethical concern related to financial compensation is that participants may be unduly influenced to join the study. As Koen *et al.* have said, inducement can be ethically justifiable, even if it contributes to participants doing something that they might otherwise not have done ^25^. Indeed, benefits in many studies are designed to encourage participation. However, they note that inducement becomes ‘undue’ where an excessive offer distorts decision-making, leading to individuals participating against their better judgment. Earlier research in Kilifi, undertaken to inform programme guidelines on study benefits, highlights a range of additional challenges that might be associated with giving high levels of benefits or payments to study participants, including commercialization of the community-researcher relationship, generating family and intra-community conflict and undermining research activities with more limited access to funding, including by the Ministry of Health itself ^22^. In the interviews, participants indicated that they did not regret joining the study. They were also aware of their right to withdraw if they felt the study was no longer suitable for them. However, we will continue assessing the impacts and implications of these levels of compensation over time, including any longer-term implications on the other studies that are conducted in the setting.

### Limitation of the study/future research ideas

This study focused on participants and study team members’ perceptions of a malaria Challenge study conducted on the Kenyan Coast. Follow up studies with a wider range of stakeholders including Ethics Review Committees (ERCs), community leaders, current and previous Challenge participants, community members and researchers will be important to explore some of the issues the current study was not able to address. This includes issues around the concept of deliberately infecting participants, implications of financial compensation on family dynamics, and alternative levels and types of benefits and compensation for CHMI studies. It would also be valuable to nest new studies in other disease Challenge studies and communities across LMICs to contextualize the emerging ethical issues and make generalizable statements on the ethical issues for CHMI studies in LMICs.

## Conclusion

There are strong reasons to conduct CHMI studies in LMICs. There is however sparse literature on ethical issues for CHMI studies in LMICs, and none of the literature has specifically explored the perceptions of participants in such studies. In addition, current ethical frameworks and guidance documents focus on clinical trials and minimal risk studies. There are currently a few recent specific guidance for studies that involve the deliberate infection of healthy volunteers in LMICs ^26, 27^. However, our research suggests that there are a myriad of ethical issues that are likely to emerge with proliferation of CHMI studies in LMICs, and that particular care is needed in ethical review to ensure that communities are not exploited. There is currently no threshold of risks and inconveniences set for more than minimal risk studies; participants could therefore bear considerably higher burdens and risks for participation in these settings than in others because of the need for the attractive offers such studies can provide. As with any research, these studies need a strong and well-considered rationale for conducting them, and place a heavy burden of responsibility on ethics review committees, funders, researchers and research organizations. The specific ethical issues related to forms and types of benefits and compensation for these types of studies also needs further discussion and investigation.

## Data availability

**Data available**: Data that may be made available include: data included in the manuscript in form of quotes; summaries of the main themes; and anonymized data transcripts of participant interviews and group discussion, in keeping with the conditions below.

**What uses are applicable**: As stipulated in the consent documents, data may be used to support any new research by other researchers in Kenya or elsewhere, where the nature of the data might be considered relevant. For data not included in the manuscript, the consent form indicates that data sharing will require the approval of the KEMRI Wellcome Trust research Programme Data Governance Committee (see below).

**Conditions under which data will be available**: Data provided in the manuscript may be used without request but with reference to the full article including the data. Other data will be made available with the approval of the KEMRI Wellcome Trust Research Programme Data Governance Committee (applications to Data_Governance_Committee@kemri-wellcome.org), only where anonymization can be adequately achieved to protect the privacy and confidentiality of the participants/respondents and any mentioned individuals and institutions, and where the proposed use is seen as relevant to the nature of the data. Where the DGC recommend this, the national KEMRI Science and Ethics Review Unit may also be asked to approve the proposed use. Conditions for data sharing are outlined in a KWTRP Data Sharing Agreement, including that:

— the requestor shall use the data only for the agreed purpose as stipulated in the application form and shall not use the data in such a way that causes damage or distress to the data subjects or communities involved in the research
— The requestor shall agree to at all times to keep the data strictly confidential, and ensure that the data users maintain confidentiality of the data
— The requestor shall not in any way attempt to seek to discover the identity of data subjects, to compromise or infringe on their privacy and confidentiality of their information.

## Footnotes

^1^Also referred to as the KEMRI C entre for Geographic Medicine, Coast (KEMRI_CGMRC)

^2^Large-scale open public meetings often convened by the local administrators (chiefs or assistant chiefs).

^3^A group of 6 experienced community facilitators lead by a Head of Community Engagement who coordinate and implement all engagement activities at the Programme

## Supplementary material

Supplementary File 1: Patient information and consent form for CHMI study by KEMRI-Wellcome Trust Research Programme (English translation).

Supplementary File 2: Test of understanding for potential CHMI participants.

Supplementary File 3: Focus Group Discussions guide for study participants.

Supplementary File 4: Interview guide for Clinical trial team.

## References

[1] Hodgson SH Juma E Salim A: Lessons learnt from the first controlled human malaria infection study conducted in Nairobi, Kenya. *Malar J.* 2015;14:182. 10.1186/s12936-015-0671-x 25927522 PMC4416324

[2] Shekalaghe S Rutaihwa M Billingsley PF: Controlled human malaria infection of Tanzanians by intradermal injection of aseptic, purified, cryopreserved *Plasmodium falciparum* sporozoites. *Am J Trop Med Hyg.* 2014;91(3):471–80. 10.4269/ajtmh.14-0119 25070995 PMC4155546

[3] Spring M Polhemus M Ockenhouse C: Controlled human malaria infection. *J Infect Dis.* 2014;209 Suppl 2:S40–5. 10.1093/infdis/jiu063 24872394

[4] Miller FG Grady C: The ethical challenge of infection-inducing challenge experiments. *Clin Infect Dis.* 2001;33(7):1028–33. 10.1086/322664 11528576

[5] Agnandji ST Huttner A Zinser ME: Phase 1 Trials of rVSV Ebola Vaccine in Africa and Europe. *N Engl J Med.* 2016;374(17):1647–60. 10.1056/NEJMoa1502924 25830326 PMC5490784

[6] Afolabi MO Tiono AB Adetifa UJ: Safety and Immunogenicity of ChAd63 and MVA ME-TRAP in West African Children and Infants. *Mol Ther.* 2016;24(8):1470–1477. 10.1038/mt.2016.83 27109630 PMC5010143

[7] Polhemus ME Magill AJ Cummings JF: Phase I dose escalation safety and immunogenicity trial of *Plasmodium falciparum* apical membrane protein (AMA-1) FMP2.1, adjuvanted with AS02A, in malaria-naïve adults at the Walter Reed Army Institute of Research. *Vaccine.* 2007;25(21):4203–12. 10.1016/j.vaccine.2007.03.012 17442466

[8] Hodgson SH Juma E Salim A: Evaluating controlled human malaria infection in Kenyan adults with varying degrees of prior exposure to *Plasmodium falciparum* using sporozoites administered by intramuscular injection. *Front Microbiol.* 2014;5:686. 10.3389/fmicb.2014.00686 25566206 PMC4264479

[9] US: National Library of Medicine. *ClinicalTrials.gov*. Reference Source

[10] Evers DL Fowler CB Mason JT: Deliberate Microbial Infection Research Reveals Limitations to Current Safety Protections of Healthy Human Subjects. *Sci Eng Ethics.* 2015;21(4):1049–1064. 10.1007/s11948-014-9579-z 25150847

[11] Hope T McMillan J: Challenge studies of human volunteers: ethical issues. *J Med Ethics.* 2004;30(1):110–6. 10.1136/jme.2003.004440 14872087 PMC1757139

[12] Rosenbaum JR Sepkowitz KA: Infectious disease experimentation involving human volunteers. *Clin Infect Dis.* 2002;34(7):963–971. 10.1086/339328 11880963

[13] Marsh V Kamuya D Rowa Y: Beginning community engagement at a busy biomedical research programme: experiences from the KEMRI CGMRC-Wellcome Trust Research Programme, Kilifi, Kenya. *Soc Sci Med.* 2008;67(5):721–33. 10.1016/j.socscimed.2008.02.007 18375028 PMC2682178

[14] Scott JA Bauni E Moisi JC: Profile: The Kilifi Health and Demographic Surveillance System (KHDSS). *Int J Epidemiol.* 2012;41(3):650–7. 10.1093/ije/dys062 22544844 PMC3396317

[15] Kamuya DM Marsh V Kombe FK: Engaging communities to strengthen research ethics in low-income settings: selection and perceptions of members of a network of representatives in coastal Kenya. *Dev World Bioeth.* 2013;13(1):10–20. 10.1111/dewb.12014 23433404 PMC3654571

[16] Molyneux CS Peshu N Marsh K: Trust and informed consent: insights from community members on the Kenyan coast. *Soc Sci Med.* 2005;61(7):1463–73. 10.1016/j.socscimed.2004.11.073 16005781

[17] Kamuya DM Molyneux CS Theobald S: Gendered negotiations for research participation in community-based studies: implications for health research policy and practice. *BMJ Glob Health.* 2017;2(2):e000320. 10.1136/bmjgh-2017-000320 29225935 PMC5717932

[18] Molyneux CS Murira G Masha J: Intra-household relations and treatment decision-making for childhood illness: a Kenyan case study. *J Biosoc Sci.* 2002;34(1):109–31. 11814209

[19] Royal College of Physicians L: Guidelines on the practice of ethics committees in medical research with human participants. London.2006 Reference Source PMC5375472 30667867

[20] CIOMs: International Ethical Guidelines for Health-related Research Involving Humans. S. Council for International Organizations of Medical, Editor. Geneva.2016;119 Reference Source 40523065

[21] Evers DL Fowler CB Mason JT: Deliberate Microbial Infection Research Reveals Limitations to Current Safety Protections of Healthy Human Subjects. *Sci Eng Ethics.* 2015;21(4):1049–64. 10.1007/s11948-014-9579-z 25150847

[22] Njue M Kombe F Mwalukore S: What are fair study benefits in international health research? Consulting community members in Kenya. *PLoS One.* 2014;9(12):e113112. 10.1371/journal.pone.0113112 25470596 PMC4254456

[23] Wertheimer A Miller FG: Payment for research participation: a coercive offer? *J Med Ethics.* 2008;34(5):389–92. 10.1136/jme.2007.021857 18448723

[24] Ballantyne A: Benefits to research subjects in international trials: do they reduce exploitation or increase undue inducement? *Dev World Bioeth.* 2008;8(3):178–91. 10.1111/j.1471-8847.2006.00175.x 19046255

[25] Koen J Slack C Barsdorf N: Payment of trial participants can be ethically sound: moving past a flat rate. *S Afr Med J.* 2008;98(12):926–9. 19374064

[26] Bambery B Selgelid M Weijer C: Ethical Criteria for Human Challenge Studies in Infectious Diseases. *Public Health Ethics.* 2016;9(1):92–103. 10.1093/phe/phv026 29731811 PMC5926904

[27] Gordon SB Rylance J Luck A: A framework for Controlled Human Infection Model (CHIM) studies in Malawi: Report of a Wellcome Trust workshop on CHIM in Low Income Countries held in Blantyre, Malawi [version 1; referees: 2 approved]. *Wellcome Open Res.* 2017;2:70. 10.12688/wellcomeopenres.12256.1 29018841 PMC5627502

