# Supplementary material for: Ethical considerations in Controlled Human Malaria Infection studies in low resource settings: Experiences and perceptions of study participants in a malaria Challenge study in Kenya
Source: Wellcome Open Res. 2018 Oct 29;3:39. Originally published 2018 Apr 11. [Version 2] doi: 10.12688/wellcomeopenres.14439.2 (PMC5954342; doi:10.12688/wellcomeopenres.14439.2)
Supplement: Supplementary file 2 [file wellcomeopenres-3-16209-s0001.tgz › 1e9075ea-ae3d-4e75-bc90-1418abcae614.pdf]

Controlled Human Malaria Infection (CHMI) to assess human immunity to P. falciparum using sporozoites administered by direct venous inoculation

TEST OF UNDERSTANDING

This questionnaire is designed to test your understanding of the study in order for us to be confident that you fully understand what taking part will involve. Please make sure you have read the information sheet in full and asked the Investigator any questions you may have. You need to answer all questions correctly in order to take part in the study. If you don't answer all the questions correctly the first time, you will be able to complete the questionnaire again after discussion with the Investigator.

Volunteer Name:……………………..Volunteer Trial Number:….……….Date……………..Time…….

Please clearly circle one answer for each question;

1. By participating in this study you can expect to develop which of the following:

A. Tuberculosis

B. Malaria

C. Typhoid

2. The study involves volunteers being given malaria by:

A. Spider bite

B. Mosquito bite

C. Injection

3. Is it likely that a single treatment course will be effective to treat malaria in this study?

A. Yes

B. No

4. Medical screening for this study will include which of the following?

A. Laboratory tests (including an HIV test)

B. Physical examination

C. Review of medical history

D. All of the above

E. None of the above

5. If you wish to withdraw from the study you may:

A. Withdraw voluntarily at any time provided you complete a course of anti-malarial therapy (if needed)

B. Withdraw from the study only if the investigators say it is ok

C. Never withdraw from the study

6. Which of the following are true regarding pregnancy and participation in this study?

A. Pregnant women may participate in this study

B. Women should not get pregnant for 12 months after getting malaria

C. An effective method of birth control is required for women while participating in this study

7. What are common symptoms associated with malaria infection?

A. Fever

B. Chills

C. Headache

D. All of the above

E. None of the above

8. How is malaria diagnosed in the study?

A. Looking at a sample of blood under a microscope

B. Chest X-ray

C. Having you walk on a treadmill

9. If you develop malaria, we will:

A. Treat you immediately with effective medications

B. See how sick you can get without treating you
