# Supplementary material for: Ethical considerations in Controlled Human Malaria Infection studies in low resource settings: Experiences and perceptions of study participants in a malaria Challenge study in Kenya
Source: Wellcome Open Res. 2018 Oct 29;3:39. Originally published 2018 Apr 11. [Version 2] doi: 10.12688/wellcomeopenres.14439.2 (PMC5954342; doi:10.12688/wellcomeopenres.14439.2)
Supplement: Supplementary file 3 [file wellcomeopenres-3-16209-s0002.tgz › 3973a187-a575-4683-8a5f-e8275cc1cf7f.docx]

**Supplementary file 3: FOCUS GROUP DISCUSSIONS (FGDS) GUIDE FOR PARTICIPANTS**

| **Familiarization with participants/Easing them into the discussion**   - How did you find out about this study? Who informed you? - What information were you given about this study? When invited to come the first day what did the caller say was going to happen? (probe for screening/consent processes) - What made you decide to come on the first day? | **Informed consent process**   - Do you feel you were given enough information during the info giving sessions? - Was the information easy to understand? - Any information you found challenging to understand? - What more information would you have wanted to get? - Were there enough chances to ask questions? - Although you are free to withdraw from the study, how do you feel about the need to stay and extra 3 days? - Why is it important to stay for the extra 3 days? |
| --- | --- |
| **Concepts of the challenge study**   - Briefly explain what is happening in this study? - Was is the study aiming to find out? - What do you think about the idea of infecting people with an illness to observe them? - How did you explain the study to your family members? | **Motivating factors**   - What are some of the reasons that made you want to participate? - What things did you consider while making the decision to participate? (Time? Procedures? Compensation? Anything else?) - Is there a point at which during this study you’ve felt like withdrawing? (if yes) Why? Thought about it but still decided to stay? Why? |
| **In-Patient settings**   - Why does the study require you to be housed at Pwani University hostels? - Any benefits you have seen about staying at the Pwani hostels? Which ones? Anything you have liked? - Any challenges faced? Anything you have disliked during your stay? - What are your feelings about not being able to leave the Pwani hostels during the study? - Have family members raised any concerns regarding your stay here? What are some of these issues/concerns?   *(Probe for: Length of time; food options; entertainment; location; rooms)* | **Experiences of participating in the study**   - What do you like most about the study? - What do you dislike most about the study? - Of all the trial activities and procedures, what was most challenging or difficult? Why? *(Bleeding/blood volumes?)* - Would you recommend your friend/family member to participate in a similar study? Why/why not? |
| **Family members/significant others**   - Who else was involved in making the decision? Why them? Anyone else? - What did you tell them regarding the study? - What reasons/explanations were given as to why they wanted to join the study to their significant others (especially for female participants) - Factors that were considered in decision making - Any concerns raised by family members regarding the study? Which ones? | **Relationship with clinical/Trial staff**   - Have you been able to raise issues/ask questions to the clinical staff? Do you feel they are available/accessible? - Do you feel that your questions have been responded to adequately/in time?   Any issues that were not responded to? Which ones? Do you still require information regarding this? |
| **Future studies**   - Would you be willing to participate in a similar study (challenge) in the future? - What if the level of compensation was less/none? - Would you be willing to participate in a different type of study in the future? Why/why not? Low/no benefits? | **Female participants**   - How did you feel about the need to use contraceptives? - What was the reason given as to why you need to use them? |
| **For those screened out in phase 1**   - What made you come this time even though you did not make it to enroll into the study the last time? - What information did you receive? - How did you feel last time when you did not make it to enroll? What information were you given about not being able to enroll into the study the last time? - What do you feel about coming back? Did you have any expectations the last time you came? |  |
