# Supplementary material for: Ethical considerations in Controlled Human Malaria Infection studies in low resource settings: Experiences and perceptions of study participants in a malaria Challenge study in Kenya
Source: Wellcome Open Res. 2018 Oct 29;3:39. Originally published 2018 Apr 11. [Version 2] doi: 10.12688/wellcomeopenres.14439.2 (PMC5954342; doi:10.12688/wellcomeopenres.14439.2)
Supplement: Supplementary file 4 [file wellcomeopenres-3-16209-s0003.tgz › 97fe7e0d-b4bd-4758-a04c-34b1adf84d29.docx]

**Supplementary file 4: Interview guide for study team**

| 1. **Background Information**  - What are your roles in the Malaria challenge study; what do you do? | |
| --- | --- |
| 1. **Concept of the challenge study**  - Briefly explain the aim of the challenge study - What do you think of the idea of infecting people with pathogens in research? - Do you think it’s a familiar concept in our setting? (*research Centre/community*) Why/why not? - Any concerns regarding this type of study? From staff/community? - Any limits as to the kind of diseases that can be studied using this method in Kilifi? What (ethical) issues can this bring? | |
| 1. **Informed consent processes**  - What have been the main concerns raised regarding the study by the participants during the study/consenting? (expected/unexpected) - Are there areas regarding the study that participants seems to have a challenge in understanding? - Are there issues that have been most challenging to discuss or respond to? How these have been dealt with? | 1. **Community Engagement**  - What information should be given to the community members regarding this type of study? - Any issues regarding the study that you think would potentially raise concerns? Which ones? - Who should be engaged before a study like this begins? - Should it be targeted (specific population) or done through mass barazas? Any potential challenges/concerns? |
| 1. **Relationships between the Clinical/Trial Staff and the participants**  - Do you feel that participants are free to raise issues with you? Have they been coming to ask questions? - Which questions were most common? Any unexpected questions? - Any efforts to make participants feel that the clinical staff are available? What efforts? - Are there issues raised that you have not been able to deal with or resolve? Trial/non-trial related? | 1. **Motivation for participating**  - Why do you think people participate in challenge type of studies? - What factors do you think motivate people to participate in such studies? - Compared to other studies, the level of benefits are higher in challenge studies due to high inconvenience – any comments/concerns regarding the level of benefits in these types of studies? - Do you feel as though people have already made the decision to participate before they come for consenting/screening? - Do you think anybody who agreed to be in the study is now regretting? What makes you say that? - For those screened out- do you feel there is a sense of disappointment? Why/ why not? |
