# Supplementary material for: Ethical considerations in Controlled Human Malaria Infection studies in low resource settings: Experiences and perceptions of study participants in a malaria Challenge study in Kenya
Source: Wellcome Open Res. 2018 Oct 29;3:39. Originally published 2018 Apr 11. [Version 2] doi: 10.12688/wellcomeopenres.14439.2 (PMC5954342; doi:10.12688/wellcomeopenres.14439.2)
Supplement: Peer review history

# Peer review: Referee response for version 1

**DOI:** 10.21956/wellcomeopenres.15717.r32933

**Published:** 2018-05-15

**Authors:** Danielle I. Stanisic

**Affiliations:** 1 Institute for Glycomics, Griffith University, Southport, Qld, Australia

This is a timely and important study given the increasing interest in conducting controlled human malaria infection studies in LMIC. It was conducted within the framework of ongoing CHMI studies at KEMRI, which has a long-standing, internationally recognised health research program. The authors aimed to identify ethical considerations that are uniquely relevant to studies that involve purposefully infecting healthy volunteers in LMIC. Data was collected via focus group discussions with study staff and an exit questionnaire.  

 Given the limited specific ethical guidance for studies that fall outside the categories of “clinical trials” and “minimal risk”, it will be important to consider the findings of this current study when designing and planning future CHMI studies. This current study also provides a framework for the conduct of similar social science evaluations within other CHMI studies. As the infrastructure for CHMI (and potentially other challenge models) are established in new communities and new LMICs, it will be of great interest to identify common and disparate ethical issues across the different populations.  

 I have a few questions and comments for the authors.

1. Introduction, 1 ^st^ paragraph, last sentence. It is worth noting that CHMI studies in non-malaria endemic countries have shown to be a useful tool for the screening of pre-erythrocytic vaccine candidates. A recent review ^1^ discusses considerations for the similar testing of blood-stage vaccine candidates. Malaria-naïve individuals in non-endemic countries are highly clinically sensitive to low levels of blood-stage parasites and thus may require drug treatment soon after challenge, prior to when vaccine efficacy may be apparent. This may result in prematurely discarding a vaccine candidate. In the presence of pre-existing Plasmodium-specific immune responses in malaria endemic areas, where clinical thresholds are known to be much higher, such a vaccine may be shown to be highly efficacious. Thus, CHMI in malaria endemic countries may also play a critical role in enabling assessment of blood-stage vaccine candidates in early and late phase trials. 
2. There is discussion throughout the document about lack of ethical guidance on studies that are beyond “minimal” risk. I may have missed it, but is it definitively stated in the introduction what minimal risk is and that these challenge studies do not meet these criteria? This would be helpful. Evers et al 2015 provide the std definition which may be useful: “Minimal risk may be defined as no greater likelihood of insult or injury than that encountered in daily life or in a routine medical or psychological examination (Council of Europe 2012; FDA and HHS regulations), or alternatively, at most, a very slight and temporary detrimental impact on the health of the research participant (Council of Europe 2005).”
3. The complex nature of CHMI studies and the risks of being involved present a challenge in terms of providing information and ensuring that the potential participants are truly able to give informed consent (particularly if there are variable levels of education). The questionnaire that was used to confirm understanding of the study is hopefully a robust tool to identify individuals who do not understand the study and can therefore not give true informed consent for participation. There is a comment in the introduction (Page 3, 2 ^nd^ column) that “researchers often target participants with higher levels of education as most likely to give informed" and the dilemma of excluding those with low formal education. It is interesting to note (and maybe this should be highlighted) that this may be unnecessarily exclusionary as at least in this current study, participants who had very low levels of schooling could also correctly answer the test of understanding questions.
4. The statement “Participants said that they were not worried about being injected with the malaria parasites, but said they would be concerned about being injected with a less familiar disease-causing organism”. It is interesting that familiarity with malaria was one of the factors that influenced participation and again, maybe this should be highlighted. If researchers are wanting to conduct studies with less familiar organisms in LMIC, this would need to be considered when designing tools for participant recruitment.
5. Was participation influenced at all by potentially finding out that they may be infected with other organisms eg HIV? I noted in the PICF, that it is stated that they will be referred to counselling if this is the case. In areas where HIV is a major issue, it would be interesting to know how much this was influencing the decision to participate. On Page 12, first column and first participant quote “FGD1 P8 That day when I was injected she came back and told me “you have already been injected with HIV””. This sounds like at least one participant was told they were infected with HIV via the blood-screening process at the start of the study.
6. On page 7, there are a lot of participant quotes indicating that the payment played an important role in the decision to participate. One participant even worried about when drug treatment was being initiated as he had already pre-allocated the full amount of monetary compensation. To fully appreciate the role of payment, it would be interesting to know if during the study, it was found that a participant had withheld information about medication, medical conditions etc that would make them ineligible to participate. I realise it is a small sample size, but was this examined?
7. On page 8, bottom of first column. There is some discussion about people not disclosing to their family that they had participated due to concerns about their reaction. Does this raise additional ethical issues given the studies are more than “minimal risk” e.g. what if there was some sort of study-related health issue that arose after they were discharged from the facility/study or if a major health issue arose during the study that would require the family to be informed? Given they were housed in the same facility – were the participants co-mingling i.e. could they identify each other and would this cause any disadvantage to those participants e.g. those who had chosen not to disclose their involvement to their family.  It would be interesting to discuss this.
8. In the discussion, page 9, first column, 3 ^rd^ paragraph. It is stated ”Participants’ concern about study safety also contributed to their active surveillance of the well-being of participants who had participated in the previous Challenge events, seeing that they were well seemed to allay some concerns”. Does this mean that they were aware of who the previous participants were or were they informed generally of participant progress by the study team?  If the former, again, does this raise additional ethical issues?

I have read this submission. I believe that I have an appropriate level of expertise to confirm that it is of an acceptable scientific standard.

## References

[1] GoodMFMillerLH: Interpreting challenge data from early phase malaria blood stage vaccine trials. *Expert Rev Vaccines*.2018;17(3) : 10.1080/14760584.2018.1435278 189-196 10.1080/14760584.2018.1435278 29382292

# Response

**Published:** 2018-10-10

**Authors:** Maureen Njue

**Affiliations:** KEMRI-Wellcome Trust Research Programme, Kilifi, Kenya, Kenya

Thank you for highlighting the important contribution our study makes. We appreciate your comments and suggestions to helping improve this manuscript. We have responded to the issues raised below.

**I have a few questions and comments for the authors.**

Introduction, 1 ^st^ paragraph, last sentence. It is worth noting that CHMI studies in non- malaria endemic countries have shown to be a useful tool for the screening of pre-erythrocytic vaccine candidates. A recent review ^1^ discusses considerations for the similar testing of blood-stage vaccine candidates. Malaria-naïve individuals in non-endemic countries are highly clinically sensitive to low levels of blood-stage parasites and thus may require drug treatment soon after challenge, prior to when vaccine efficacy may be apparent. This may result in prematurely discarding a vaccine candidate. In the presence of pre-existing Plasmodium-specific immune responses in malaria endemic areas, where clinical thresholds are known to be much higher, such a vaccine may be shown to be highly efficacious. Thus, CHMI in malaria endemic countries may also play a critical role in enabling assessment of blood-stage vaccine candidates in early and late phase trials. 

**Response:**

**We agree that vaccine responses and protective efficacy may be different in adults in malaria endemic settings, and that this is of scientific interest, in addition to studies of naturally acquired immunity as done here.** 

There is discussion throughout the document about lack of ethical guidance on studies that are beyond “minimal” risk. I may have missed it, but is it definitively stated in the introduction what minimal risk is and that these challenge studies do not meet these criteria? This would be helpful. Evers et al 2015 provide the std definition which may be useful: “Minimal risk may be defined as no greater likelihood of insult or injury than that encountered in daily life or in a routine medical or psychological examination (Council of Europe 2012; FDA and HHS regulations), or alternatively, at most, a very slight and temporary detrimental impact on the health of the research participant (Council of Europe 2005).”

**Response:**

**Thank you for this advice and we have included the definition of minimal risks in the discussions section (page 9)** 

The complex nature of CHMI studies and the risks of being involved present a challenge in terms of providing information and ensuring that the potential participants are truly able to give informed consent (particularly if there are variable levels of education). The questionnaire that was used to confirm understanding of the study is hopefully a robust tool to identify individuals who do not understand the study and can therefore not give true informed consent for participation. There is a comment in the introduction (Page 3, 2 ^nd^ column) that “researchers often target participants with higher levels of education as most likely to give informed" and the dilemma of excluding those with low formal education. It is interesting to note (and maybe this should be highlighted) that this may be unnecessarily exclusionary as at least in this current study, participants who had very low levels of schooling could also correctly answer the test of understanding questions. 

***Response:***

**Thank you for this very useful comment. We have added a sentences in the discussion section (page 9) to reflect the suggestion but also a caution that it could be that education standards might be important in some types of studies. In our case, malaria is fairly common and people are familiar with it. However some of the elements of the study are unfamiliar (e.g. deliberately being infected). An emphasis we have highlighted in the discussion section is the importance of careful and well thought-out information giving sessions and community engagement.**

The statement “Participants said that they were not worried about being injected with the malaria parasites, but said they would be concerned about being injected with a less familiar disease-causing organism”. It is interesting that familiarity with malaria was one of the factors that influenced participation and again, maybe this should be highlighted. If researchers are wanting to conduct studies with less familiar organisms in LMIC, this would need to be considered when designing tools for participant recruitment. 

***Response:***

**We agree. When working with less familiar organisms/pathogens, concerted efforts should be made to create awareness about the resulting illness from the pathogens to potential participants and communities. In addition, participants should be provided with time to potentially consult widely, and allow people to participate based on the knowledge and whether they feel comfortable to participate in a study that involves that kind of organism. In this case, the test of understanding was a useful tool.**

Was participation influenced at all by potentially finding out that they may be infected with other organisms e.g. HIV? I noted in the PICF, that it is stated that they will be referred to counselling if this is the case. In areas where HIV is a major issue, it would be interesting to know how much this was influencing the decision to participate. On Page 12, first column and first participant quote “FGD1 P8 That day when I was injected she came back and told me “you have already been injected with HIV””. This sounds like at least one participant was told they were infected with HIV via the blood-screening process at the start of the study. 

***Response:***

**The benefits of getting a full body ‘health check’ from the screening process was one of the important motivating factors for most of the participants, however, there was no specific mention about HIV screening being particularly appreciated. Within the study context, HIV testing and counselling services are offered free of charge at public health Institutions. There seemed to be greater mention of the other tests which would be expensive to do in public and private clinics/hospitals e.g. checking the heart, the liver etc.**

**For the participant in FGD1 P8 – The conversation was around the general mistrust around research and research scientists’ intentions. The participant shared with a friend about her research participation in the malaria study and that they were injected with malaria parasites. The participant’s friend raised questions around the uncertainty of whether the injection was actually malaria or something else such as HIV which is more dangerous and without cure.  In the discussion section, we discuss the importance of careful community engagement activities that provide a platform for community members and research participants (page 9). This allows them to raise issues and concerns that can be addressed to clear any misunderstandings and misconceptions about on-going research and research activities in general.**

On page 7, there are a lot of participant quotes indicating that the payment played an important role in the decision to participate. One participant even worried about when drug treatment was being initiated as he had already pre-allocated the full amount of monetary compensation. To fully appreciate the role of payment, it would be interesting to know if during the study, it was found that a participant had withheld information about medication, medical conditions etc that would make them ineligible to participate. I realise it is a small sample size, but was this examined? 

***Response:***

**Based on our findings, there was no indication that people withheld information regarding their health to gain entry into the study. The screening processes also checked for recent use of medications.  However, this is an area we are keen to continue monitoring as more CHMI studies are conducted in our settings, including findings from an on-going social science exploring social implications of involvement for healthy volunteers in CHMI studies. We hope that the on-going study will shed more light on some of these other issues that this study was not able to explore.**

On page 8, bottom of first column. There is some discussion about people not disclosing to their family that they had participated due to concerns about their reaction. Does this raise additional ethical issues given the studies are more than “minimal risk” e.g. what if there was some sort of study-related health issue that arose after they were discharged from the facility/study or if a major health issue arose during the study that would require the family to be informed? Given they were housed in the same facility – were the participants co-mingling i.e. could they identify each other and would this cause any disadvantage to those participants e.g. those who had chosen not to disclose their involvement to their family.  It would be interesting to discuss this. 

***Response:***

**The participants were invited from 3 locations in Kilifi and during the study, all participants were housed at the same boarding facility and spent time together during their stay. Some of the participants knew each other before joining the study since they had been recruited from the same localities.**

**The finding that some people had not fully disclosed to their families or significant others that they were involved in the CHMI study was interesting and unexpected. We were not able to follow-up the implications of this non-disclosure of information but on-going social science work in Kilifi is currently exploring this in more depth.**

In the discussion, page 9, first column, 3 ^rd^ paragraph. It is stated ”Participants’ concern about study safety also contributed to their active surveillance of the well-being of participants who had participated in the previous Challenge events, seeing that they were well seemed to allay some concerns”. Does this mean that they were aware of who the previous participants were or were they informed generally of participant progress by the study team?  If the former, again, does this raise additional ethical issues? 

***Response:***

**Confidentiality and privacy is emphasized by the trial team during the informed consent processes, but there’s anecdotal information that individuals participating in clinical studies often share information about their own participation with others either in their families or with friends. In this study, some participants mentioned that they were advised by others who had participated in the previous cohort to join the next study. See excerpts from the FGDs  below (these are not included in the manuscript):**

***[FGD3:P5: Among those who came in the last phase…when they came back they looked for us and told us that it would have been better if we had accepted, that we were lied to [by those spreading rumours]. All the same there are other phases coming if we choose to join that’s fine, if we don’t then it’s up to us.]  ( FGD with men)***

***[FGD4 P8: I was not worried because I also had my wife who participated last year.  I knew there were no risks or worries that you were being injected with a disease or whatever.  Not at all, in fact I was so happy.] (FGD with men)***

# Peer review: Referee response for version 1

**DOI:** 10.21956/wellcomeopenres.15717.r32994

**Published:** 2018-05-14

**Authors:** Christine Grady

**Affiliations:** 1 Clinical Center, Department of Bioethics, National Institutes of Health, Bethesda, MD, USA

Thank you for the opportunity to review this interesting manuscript. Acknowledging that controlled human infection or challenge studies are increasingly being proposed or conducted in LMIC, these authors set out to better understand some of the ethical issues by exploring the perspectives and experiences of volunteers in a malaria challenge study in Kenya. They found that most of their respondents had a reasonable understanding of key elements of the study and were motivated primarily by the financial compensation. Respondents expressed concerns about the amount of blood drawn and about how this kind of research might affect public views of research in general.

 The topic is clearly relevant and timely, as more CHIM studies are being proposed or conducted in LMIC. The study methodology used was appropriate and described clearly, the research team is experienced and very good at this type of research. In their methods, they describe observation as well as focus groups with research participants and in-depth interviews with two staff members. In the results, they report themes that emerged including the informed consent process, participant perceptions of the study and infection model, motivations for participation, negotiating participation with significant others, and participant experiences. They illustrated each of these themes with quotes from one of the focus group participants. They divide their discussion into risks and burdens, community engagement and consent, and levels of compensation. 

 Overall, I think this is an important and well-described study. I have a few questions and suggestions for improving the manuscript. First, I could not tell whether they report any results of the in-depth interviews with staff or of the observation, the results all seem to be from focus group participants. Is that the case? Were there any insights from the staff member interviews? Second, I wonder if the authors could comment on the influence of gender. The majority of volunteers in the CHIM study was male. Yet, 14 of the 15 female challenge study participants participated in focus groups and many fewer of the male participants (22/49). Female participants had less education than the males. In reported results, quotes (12 v. 7) were more often from the female focus group participants than from males. Please comment on this.  

 The first section of the discussion is about risks and burdens in challenge studies, I agree that it may be the most important ethical issue to consider. Yet, it was interesting to me that in the reported results, the participants seemed more concerned about blood draws than about being injected with malaria. Is that correct? Could this discrepancy be addressed?

 Lastly, the authors conclude that there is a need for specific guidance or frameworks for CHIM. Two recent papers offer some guidance (in addition to the papers that they already cited): 1) Bambery et al. (2016) ^1^ and 2) Gordon et al. (2017) ^2^.

I have read this submission. I believe that I have an appropriate level of expertise to confirm that it is of an acceptable scientific standard.

## References

[1] BamberyBSelgelidMWeijerCSavulescuJPollardAJ: Ethical Criteria for Human Challenge Studies in Infectious Diseases. *Public Health Ethics*.2016;9(1) : 10.1093/phe/phv026 92-103 10.1093/phe/phv026 29731811PMC5926904

[2] GordonSBRylanceJLuckAJamboKFerreiraDMManda-TaylorLBejonPNgwiraBLittlerKSeagerZGibaniMGmeinerMRoestenbergMMlombeYWellcome Trust CHIM workshop participants: A framework for Controlled Human Infection Model (CHIM) studies in Malawi: Report of a Wellcome Trust workshop on CHIM in Low Income Countries held in Blantyre, Malawi. *Wellcome Open Res*.2017;2: 10.12688/wellcomeopenres.12256.1 70 10.12688/wellcomeopenres.12256.1 29018841PMC5627502

# Response

**Published:** 2018-10-10

**Authors:** Maureen Njue

**Affiliations:** KEMRI-Wellcome Trust Research Programme, Kilifi, Kenya, Kenya

Thank you for highlighting the important contribution our manuscript makes and we are grateful for the comments and suggestions offered to improve this manuscript. We have responded to the issues raised below.  

**Question**:

Overall, I think this is an important and well-described study. I have a few questions and suggestions for improving the manuscript. First, I could not tell whether they report any results of the in-depth interviews with staff or of the observation, the results all seem to be from focus group participants. Is that the case? Were there any insights from the staff member interviews?

***Response:***

**The social science study utilized observations, Interviews and Focus Group Discussions (FGDs). The observations were mainly around the informed consent processes and participant engagement activities. The information gathered provided the researcher with insights into the clinical trial activities and facilitated the development of the interviews and FGDs question guides and supported analysis. A statement has been added to illustrate the use of the observation data (Page 4).**

**The interviews were conducted with only two staff members in the study.  In the analysis, insights from the staff were mainly in agreement with the findings from the participants in the FGDs. We have now added a few illustrative quotes from the study team into the manuscript (Page 6 and 8).**

**Question**:

Second, I wonder if the authors could comment on the influence of gender. The majority of volunteers in the CHIM study was male. Yet, 14 of the 15 female challenge study participants participated in focus groups and many fewer of the male participants (22/49). Female participants had less education than the males. In reported results, quotes (12 v. 7) were more often from the female focus group participants than from males. Please comment on this.  

***Response***

**Although we had not set out to explore gender related issues in the study, we identified a few gender differences in the analysis. These differences are briefly mentioned in different sections of the manuscript, mainly in the results section, although they were not pooled together since, a discussion on gender related issues was beyond the scope of the current study. Some of the areas we have mentioned gender-related issues are around, decision making for research participation, family and child care responsibilities for women, use of contraceptives by women while in the study and the more positive views around  being in-patient at the facility compared to the men. We however, hope to interrogate these issues further in subsequent planned social science work in this setting.**

**In the manuscript, it seems that the quotes by women were more than those of the males Whilst we had many quotes across the FGDs, we selected quotes that best illustrated the findings; and may inadvertently ended up with more female quotes than those of the males. Subsequent studies on this area that are looking at gender issues in CHMI studies will provide more information on this area.**

**Question:**

The first section of the discussion is about risks and burdens in challenge studies, I agree that it may be the most important ethical issue to consider. Yet, it was interesting to me that in the reported results, the participants seemed more concerned about blood draws than about being injected with malaria. Is that correct? Could this discrepancy be addressed?

***Response:***

**This is a very good point, which we also noted in our findings (page 6: Participants perception of the study and infection model) and in our discussion. There are several reasons we think that the participants may have had more concerns about the blood draws than the risk of getting malaria as a result of being injected with the pathogens. We suppose that the regularity and discomfort/pain of regular blood draws were more visible and acutely felt by every participant in the study; and that might have contributed to how these were discussed in the FGDs. With regards to malaria, however, the study was conducted in a malaria endemic area where there is high chance that those participating in the CHMI study may have experienced malaria at one point in their lives, and so they knew what it would feel like if one did get sick. In addition, the fact that they were closely monitored for malaria and immediately treated if found to have malaria, may have in way (we think) allayed their concerns about the disease; and their perceptions of the risks associated with it. A subsequent study has explored this area in detail and the findings will be published separately.**

Lastly, the authors conclude that there is a need for specific guidance or frameworks for CHIM. Two recent papers offer some guidance (in addition to the papers that they already cited): 1) Bambery et al. (2016) ^1^ and 2) Gordon et al. (2017) ^2^.

**Thank you for the references. We have revised the conclusion section to acknowledge the availability of a few guidelines and frameworks with which to evaluate CHMI studies in LMICs.**
